# Interpolation of Point Prevalence Rate of the Highly Pathogenic Avian Influenza Subtype H5N8 Second Phase Epidemic in South Korea

**DOI:** 10.3390/vetsci9030139

**Published:** 2022-03-16

**Authors:** Saleem Ahmad, Kye-Young Koh, Jae-il Lee, Guk-Hyun Suh, Chang-Min Lee

**Affiliations:** 1 Veterinary Public Health Lab, College of Veterinary Medicine, Chonnam National University, Gwangju 61186, Korea; 186762@jnu.ac.kr (S.A.); ggy5255@naver.com (K.-Y.K.); jaeil@jnu.ac.kr (J.-i.L.); 2 Department of Veterinary Internal Medicine, College of Veterinary Medicine and BK21 FOUR Program, Chonnam National University, Gwangju 61186, Korea; ghsuh@chonnam.ac.kr

**Keywords:** highly pathogenic avian influenza, prevalence, predictions, inverse distance weighting, kriging

## Abstract

Humans and animals are both susceptible to highly pathogenic avian influenza (HPAI) viruses. In the future, HPAI has the potential to be a source of zoonoses and pandemic disease drivers. It is necessary to identify areas of high risk that are more vulnerable to HPAI infections. In this study, we applied unbiased predictions based on known information to find points of localities with a high probability of point prevalence rate. To carry out such predictions, we utilized the inverse distance weighting (IDW) and kriging method, with the help of the R statistical computing program. The provinces of Jeollanam-do, Gyeonggi-do, Chungcheongbuk-do and Ulsan have high anticipated risk. This research might aid in the management of avian influenza threats associated with various potential risks.

## 1. Introduction

Avian influenza (AI) viruses are of enormous concern as poultry infections, not only because of their negative effects on wildlife conservation and economics, but also because they are potential zoonoses and drivers of future pandemic diseases. Viruses can be transmitted from wildlife to poultry as well as to humans. HPAI lineages can develop from low pathogenic avian influenza viruses, causing severe disease and significant morbidity and fatality in vulnerable poultry species [1].

In many countries of the globe, highly pathogenic avian influenza-H5N1 has already become widespread in the migratory birds, with wild birds transmitting the virus. The influenza viruses’ initial biological hosts, ducks, shorebirds, and gulls, are thought to have carried the virus. Infected dabbling ducks exhibit no clinical indications of infection and are thought to be carriers in the wild, but infected diving ducks are more inclined to morbidity and mortality [2].

Mandarin ducks are endemic to North as well as South Korea, China, eastern Russia in the East Asian–Australasian flyway, and also Japan. Some mandarin ducks are native to South Korea, while some are passing migrants who spend the winter in Korea and migrate to and from southern Japan [3]. Various HPAI H5 infections were detected in mandarin ducks in South Korea [4] in December 2014; clade 2.3.4.4 subgroup A H5N8 was identified [5].

Previous research studies related to 2.3.4.4 H5Nx viruses in mandarin ducks revealed that these ducks could spread the virus significantly without indicating any significant clinical manifestations of the virus infection. Moreover, these ducks then propagate the virus to other ducks through contacts, suggesting that they can end up serving as healthy carriers of clade 2.3.4.4 H5Nx virus infections [6,7,8]. Since the mandarin duck is not really a long-distance migratory bird, other long-distance migrants are suspected of serving as intermediary vectors for wider propagation [9].

After an epidemic that occurred in South Korea in January 2014 in poultry species, the H5N8 viruses propagated significantly around the globe in 2014–2015, with long-distance migrating birds playing a significant role in the global transmission of these viruses [10].

After the occurrence of HPAI H5N8 viruses at the start of 2014, these viruses were again propagated throughout the country due to the role of migratory waterfowl [5]. In Korea, sporadic outbreaks of HPAI have emerged since 2014.

Experimental research revealed that ducks infected with H5N8 had more viral replication and shedding than ducks infected with H5N1 [6]. In comparison to originally reported H5N1 HPAIVs, H5N8 demonstrated decreased cytotoxicity and modes of transmission in poultry species [11]. The experimental pathogenic properties of H242/20 (H5N8), which are comparable to Bu-an2/14(H5N8), suggest that there is potential for persistent epidemics in the absence of active surveillance and effective field countermeasures. These virulent characteristics of the HPAI virus in ducks may assist their function as a field silent carrier. A Bayesian phylodynamic study of HPAI H5N8 viruses outbreaks demonstrated that HPAI viruses first spread from migrating waterfowl to domesticated duck poultry, and then domesticated ducks mostly ended up contributing to viral propagation to chicken poultry and other smaller poultry during 2020–2021 [12].

These H5N8 virus attributes may result in late detection of HPAI symptoms and enhance the likelihood of viral spread into poultry farms. However, the mode of transmission of the HPAIVs is not yet known. More research is needed on risk variables in terms of poultry farm sanitation and viral propagation ecology in migratory and indigenous species [13].

Song et al. [14] reported that our observations in ducks are comparable with the H5N8 HPAI viruses from the 2014–2016 outbreak. One of the causes for prolonged outbreaks might be an abundance of viral shedding in ducks lacking clinical manifestations. Furthermore, genetic study revealed that H5N8 viruses in the 2014–2016 outbreak were most likely transmitted from wild birds to domesticated ducks, which played a critical role in viral propagation to domesticated poultry [15,16]. A recent research study in the Netherlands on the comparable toxicity of HPAI H5 viruses also revealed that viral shedding by duck species, including both domesticated and wild ducks, potentially promotes propagation to the poultry industry [17]. The observations in ducks, together with prior findings in Korean HPAI outbreaks, suggest that domesticated ducks contribute to the spread of HPAI H5N8 virus infections in the environment.

Between 2003 and 2015, Korea faced four highly pathogenic avian influenza H5N1 epidemics—in 2003–2004, 2006–2007, 2008, and 2010–2011. Moreover, the HPAI H5N8 outbreak that occurred during 2014–2016 was the longest, lasting four waves [18]. H5N8 HPAIV was reported in 38 wild birds and 200 poultry farms in South Korea in 2014. Despite the fact that the number of epidemic cases has decreased tremendously, the HPAIV H5N8 is still being found in poultry farms on an irregular basis. However, just a few phylogenetic analyses from this virus has been reported [19]. During 2013–2014, the H5N8 clade 2.3.4.4b HPAI virus was initially found in domestic ducks in eastern China, and a fifth epidemic was later recorded in South Korea. HPAI H5N8 clade 2.3.4.4 propagated to various countries e.g., Asia, North America and Europe, and a novel strain of H5Nx clade emerged between 16 January and 8 May 2014 in three successive phases [19]. The infecting AI viruses were identified as new reassorted influenza A (H5N8) viruses from clade 2.3.4.6, which was later renamed clade 2.3.4.4 [14,20].

South Korea, given its location on the East Asian–Australasian flyway, acts as a shelter habitat and stopping point for wild migrating birds [21,22,23]. The bodies of water near the stream located at Cheongmi, from which the 551–4/2020 virus was detected and isolated, act as a wintering site for wild migrating Anatidae birds such as mallard, spot-billed duck, but also common teal [24]. Flocks of mandarin ducks as well as spot-billed ducks were observed during sample collection at sites near Cheongmi Stream [9].

Based on the literature related to the research, it is expected that the recurrence of HPAI epidemics in the future is inevitable. Previous research studies have highlighted the dynamic behavior of HPAI virus spread, along with the variables that affect the radius of bird culling around the infected zone during the local transmission of avian influenza viruses, although there was a disparity in focusing on the areas that are at high risk. It is crucial to assess high-risk sites based on prior HPAI H5N8 epidemics [25,26]. Inverse distance weighting and kriging approaches were used to predict high-risk locations in South Korea.

According to Burrough, 1986 [27], the method of approximating the value of variables at unsampled locations within the range covered by existing point data or information is known as interpolation. Interpolation processes are frequently employed in research, particularly in fields involving geographical information and continuous processes that may be represented on a continuous spatial plane. Interpolation employs precise and descriptive summary statistics to provide a continuous representation of the event under consideration. The precision of the information used for interpolation has a direct impact on the outcome [28]. Kriging interpolation is a geostatistical approach that is frequently used in data science and external variable predictions because it delivers the best unbiased linear predictions of intermediate levels. The primary idea behind kriging interpolation is to look for typical data uniformity and anticipate regionally based values of a variable [29].

In this paper, we applied IDW and kriging interpolation methods to predict the unknown values for geographically related data points (HPAI viral risk) based on the range of a discrete set of known data points. The study findings revealed that Damyang-gun, Jeollanam-do province, with some areas of Sunchang-gun, Jeollabuk-do, Uijeongbu-si, Gyeonggi-do province as well as the areas along the borderline where Gyeongsakbuk-do, Ulsan and Gyeongsangnam-do provinces converge, should apply specific biosecurity measures. The study highlights the predicted high-risk areas that may help to control HPAI epidemics in the future.

## 2. Materials and Methods

The South Korean animal and plant quarantine agency (APQA) has released an “Epidemiology report for outbreak of highly pathogenic avian influenza (HPAI) in Republic of Korea”, which contains detailed information on avian influenza outbreaks, including poultry bird infections, bird culling and poultry farm mortality rates. The HPAI H5N8 epidemic occurred in four phases. During first phase, from 16 January 2014 to 29 July 2014, 212 outbreak cases occurred. During second phase, from 24 September 2014 to 10 June 2015, 162 cases occurred. In the third phase of the epidemic, from 14 September 2015 to 11 November 2015, 17 cases occurred. Only 2 cases occurred from 23 March 2016 to 5 April 2016, phase four of the HPAI H5N8 epidemic. We focused on the second phase of the epidemic because phase 3 and phase 4 had very few numbers of outbreak cases. We used inverse distance weighting (IDW) and kriging techniques to predict high-risk areas [30,31]. The dataset containing H5N8 outbreak cases during phase 2 is available in Supplementary Materials (Table S1).

Case definition: Soon after the discovery of poultry with clinical manifestations of HPAI infectious disease by livestock owners, farm laborers, or veterinarians, the incident must always be reported to the Animal and Plant Quarantine Agency (APQA), Gimcheon, Korea, for passive surveillance, because it is required by the law on preventative measures against highly infectious diseases. Government veterinarians went to the notified poultry farms and collected specimens from ill or deceased poultry, which were tested for HPAI infection [25,32], whereas if a suspected farm reported the presence of HPAIV, it was deemed an infected location.

All the analyses were performed in R statistical software for statistical computing step by step [33]. The R code (R.Script.txt) for all of the steps performed is available in supplemental materials (Algorithm S1).

### 2.1. Inverse Distance Weighting (IDW)

Ikechukwu et al. [34] proved that IDW strategy implies that the value at an unknown place may be estimated as a weighted average of data values inside a certain cut-off range, or from a defined number of the nearest locations (normally 10 to 30). Weights are typically inverse to a power of distance [35], resulting in an estimate for an un-sampled site as shown in Equation below (1).

(1) $$F\left(s\right)=\overset{n}{\underset{i=1}{\sum }}\;w_{i}z\left(s_{i}\right)=\frac{\frac{\sum_{i=1}^{m}\;z\left(s_{i}\right)}{{\left|s-s_{i}\right|}^{p}}}{\sum_{j=1}^{m}\frac{1}{{\left|s-s_{j}\right|}^{p}}}$$

In the above equation, ‘*p*’ is a parameter, typically equal to 2 [36]. IDW is a simple and widely available approach; nevertheless, it usually fails to construct the local shape predicted by data and instead generates localized extreme values at the sample points [36]. Some changes have resulted in a class of multivariate composite IDW landscapes and volumes [35]. The hypothesis for IDW would be that sampled points nearer to the unknown point have results that are more similar to it than those that are farther off. The weight is stated as follows:

(2) $$\lambda_{i}=\frac{\frac{1}{d_{i}^{p}}}{\sum_{i=1}^{n}\frac{1}{d_{i}^{p}}}$$

In Equation (2), d_i_ is the distance between *X*_0_ and *X_i_*, *p* stands for a parameter called ‘power’, and *n* denotes the number of sample points utilized in the prediction. The value of the power variable [37] is the most important element influencing IDW accuracy. Weights decrease as distance increases, specifically as the power variable value goes up; thus, close observations have a greater weight and have more influence on the prediction, and the resulting spatial interpolation is localized [37]. The power parameter and the neighboring zone are chosen at random [38]. The most common *p* value is 2, and the resulting approach is typically referred to as inverse distance squared ‘IDS’. The power variable can also be based primarily on inaccuracy measurements (for example, minimal mean absolute error), resulting in optimum IDW [39]. The smoothing of the predicted surface changes proportionally with the power variable, and it is determined that when *p* is 1 and 2, the estimated results are less good than when *p* is 4 [40]. IDW is referred to as a “moving average” when *p* is zero [41], “linear interpolation” in case *p* is 1, and “weighted moving average” in case *p* is not equal to 1 [42].

Using R programming [33], IDW prediction analysis was performed. In the first step, point prevalence data for HPAI H5N8 were loaded in R programming software for statistical computing. Before starting the analysis, important packages to successfully carry out the analyses were installed and loaded into R. The packages were “Metrics” [43], “spatstat” [44], “raster” [45], “sp” [46], “geoR” [47], “gtools” [48], “lme4” [49], “leaflet” [50], “oro.nifti” [51], and wesanderson [52]. In the second step, using the raster package, which offers access to GADM data, a South Korea Adm 1 level boundary file was downloaded into R.

In the third step, the known point prevalence rate of HPAI H5N8 was depicted on the map using leaflet.

In the fourth step, a window to which our predictions/interpolations were limited was created.

In the fifth step, using “spatstat” package, based on estimations of kernel density, we generated a point pattern “ppp” object with the purpose of interpolating the result as markings. First of all, we set the observation window for the ppp function, for which the owin function was used. Next, we used the bounding box around data collection sites in South Korea State. Then, a ppp of the point prevalence data was defined.

The IDW method essentially takes each of the data points, looks at all of the neighboring points, and averages the values of point prevalence taken at those neighboring points. Finally, it applies a weight to each of those averages, and comes up with a final weighted average. Those final weighted averages represent the interpolated values. The weight applied to each of those averages is determined by what is called a power function. There can be a wide range of power functions. Most importantly, the higher the power, the more weight assigned to closer neighbors of a given point [51]. Different power functions were examined, and an optimal power to run the IDW function was chosen.

In the sixth step, different IDW results were plotted with different powers using power function. Function ‘at’ in the R programming code can be ‘pixels’, which creates estimates over a grid of pixels, and which uses leave-one-out cross-validation to interpolate values at each point. To calculate the ‘best’ power to use, cross-validation was used. When calling the IDW function with the option at = points, this was achievable. We could not find an off-the-shelf code to handle this, so we used cross-validation to cycle through multiple powers and choose the one with the lowest error. Observed prevalence was plotted with predicted prevalence (expected with optimal power).

### 2.2. Kriging

According to Ikechukwu et al. [34], kriging is based on the idea of random processes, with surface or volume considered to be one implementation of a random process with already measured spatial covariance [36]. Regionalized variable theory states that the geographical variability of any variable may be described as the summation of the below listed three factors:

1. A structural component with a fixed mean or pattern.
2. A regionalized variable that is random yet geographically associated element.
3. A noise or residual component that is random yet spatially uncorrelated.

The equation for a random variable *z* at *x* can be expressed mathematically as:

(3) $$Z\left(x\right)\;=\;m\left(x\right)\;+\;\varepsilon^{\prime }\left(x\right)\;+\;\varepsilon^{\prime \prime }$$

From the above Equation (3), $m\left(x\right)$ denotes a structural feature that models the structural element, while $\varepsilon^{\prime }\left(x\right)$ denotes spatially auto-correlated stochastic residual from $m\left(x\right)$, which is the regionalized variable, and $\varepsilon^{\prime \prime }$ stands for random noise including an average of zero and a variance of σ^2^ [42].

With the help of R programming language [33], starting from the seventh step after IDW, the “GeoR” package was used to perform kriging. With the help of package “GeoR”, a geodata object was created using a data frame of x,y coordinates and point prevalence data. Using the Lowes parameter function, a summary plot of prevalence was created. The Lowes option generates Lowes curves for the x–y coordinate relationship.

In the eighth step, to observe the outcome after having regressed prevalence against x and y using a linear and polynomial effect, “trend = 2nd” to the plot command was added. A variogram was generated and plotted. We solely focused on whether there was a correlation between lower semi-variance values and lower distance classes, and vice versa, to indicate spatial autocorrelation. By binning distances into “distance classes” and utilizing arbitrary cutoff points for distances, we simplified the vario cloud. The bins used were reduced to half the maximum interpoint distance following the rule of thumb, in order to ensure that the distance classes were large/small enough for each class to have a minimum number of distances to display on a graph variogram estimation. Bin points were categorized based on their distance. The variogram bins were constructed using the “uvec” argument, which provided values to define the variogram binning. We tried bins of 0.2 decimal degrees, about 22 km, and looked at the numbers in each bin.

The variogram model was fitted with minimized least squares utilizing several covariance models in the ninth step. In this case, we used a ‘spherical’ and ‘exponential’ model, and the results were plotted. Additionally, the summaries of the fit were extracted.

In the tenth step, we utilized a variogram approach to krig results at predicted sites, which depicted the correlation between node pairs as a function of distance among coordinates. To compare with the IDW, a preliminary grid of points was produced from the IDW. After the prediction grid was created, kriging to those points was carried out. Predictions were finally visualized. After this, we created a raster of our prediction using the rasterFromXYZ function.

**Figure 1:**
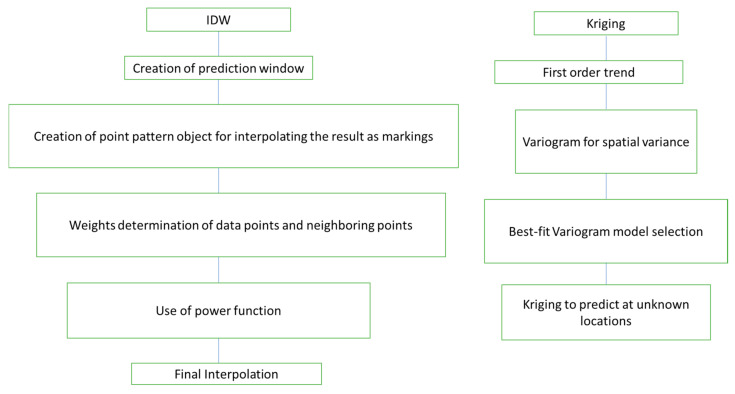
The overall scheme for the IDW and kriging analyses in this study.

Using the xvalid function in package “geoR” to generate cross-validated predictions was straightforward. We used the default leaving-one-out cross-validation for all points and then displayed the findings on a log odds scale. Final kriged predictions using leaflet map were also generated. Mean squared error (MSE) values were calculated for both the methods used for predictions. Locations where predictions from IDW differed from kriging were visualized. In the eleventh step, the known prevalence as well as the predicted prevalence values were plotted on the map using leaflet. The overall scheme for the IDW and kriging analyses is shown in Figure 1.

## 3. Results

### Descriptive Analysis

**Figure 2:**
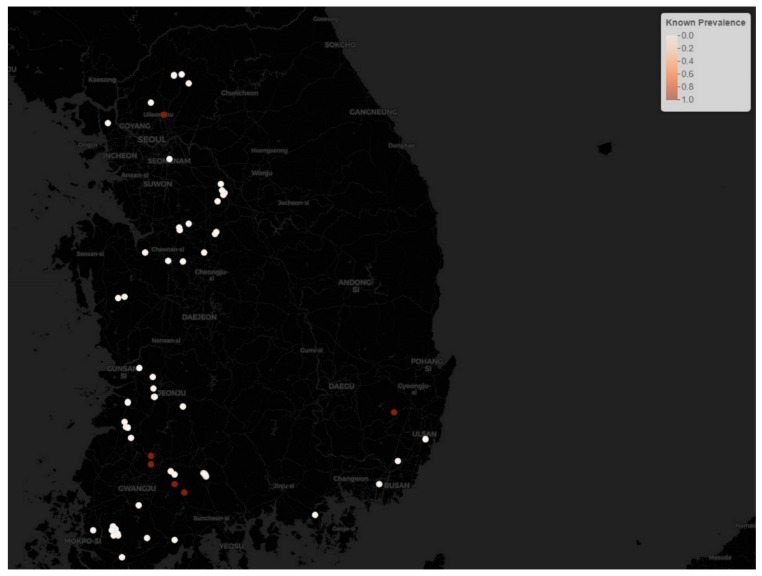
The known point prevalence rate of HPAI H5N8 epidemic phase 2. Brown dots denote high prevalence while white circles denote low prevalence.

During the second phase of the H5N8 outbreak, there were 162 cases from 24 September 2014 to 10 June 2015, for a total of 260 days. During this phase, the epidemic affected 117 (72.2%) ducks, 39 (24.1%) chickens, and 6 (3.7%) other species such as quails and ostriches. The number of cases per day out of total susceptible birds as a point prevalence rate of HPAI H5N8 in each poultry farm was computed and is visualized in Figure 2.

**Figure 3:**
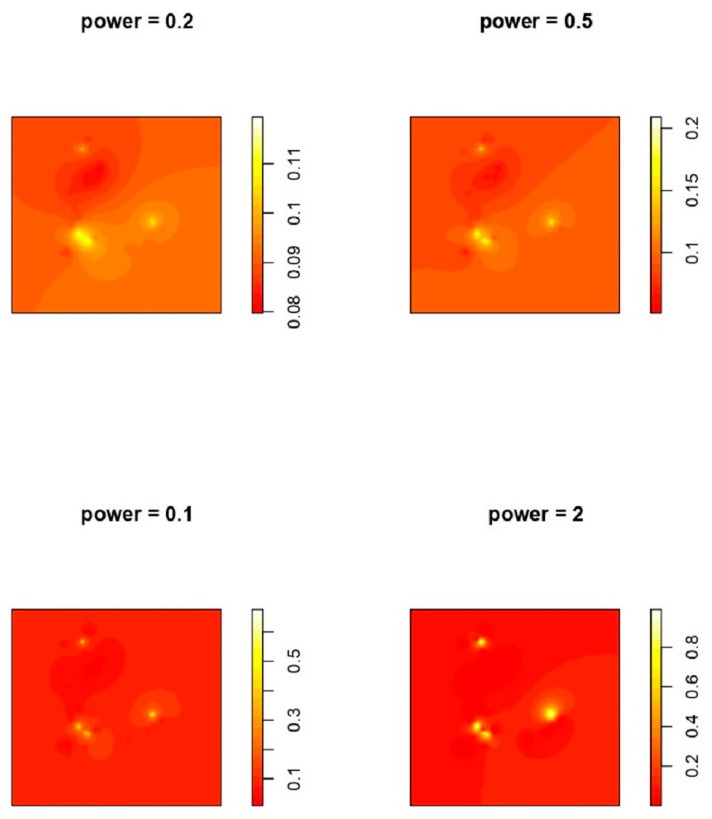
IDW interpolation with different powers. Power indicates the power function.

We assumed that things that are nearer together are more similar than those that are farther away. According to IDW, every measurable location has a local impact that decreases with distance. We applied IDW to predict a point prevalence rate for HPAI epidemics for every undefined point by using the measured data surrounding the prediction location. Interpolation assigns more weight to points nearest to the prediction point, and the weights decrease as distance increases [53]. Weights are proportional to the inverse of the distance (between the data point and the predicted location) raised to the value of power ‘p’. Consequently, as the distance increases, the weights rapidly drop. The pace at which the weights decrease is determined by *p*. If *p* = 0, there is no reduction with distances, and the prediction is the mean of all the data values in the search window since each weight I is the same. The weights for remote locations suddenly fall as *p* rises. If the *p* value is quite high, the prediction will be influenced primarily by the points closest around it. Results of IDW are depicted with different powers in Figure 3.

**Figure 4:**
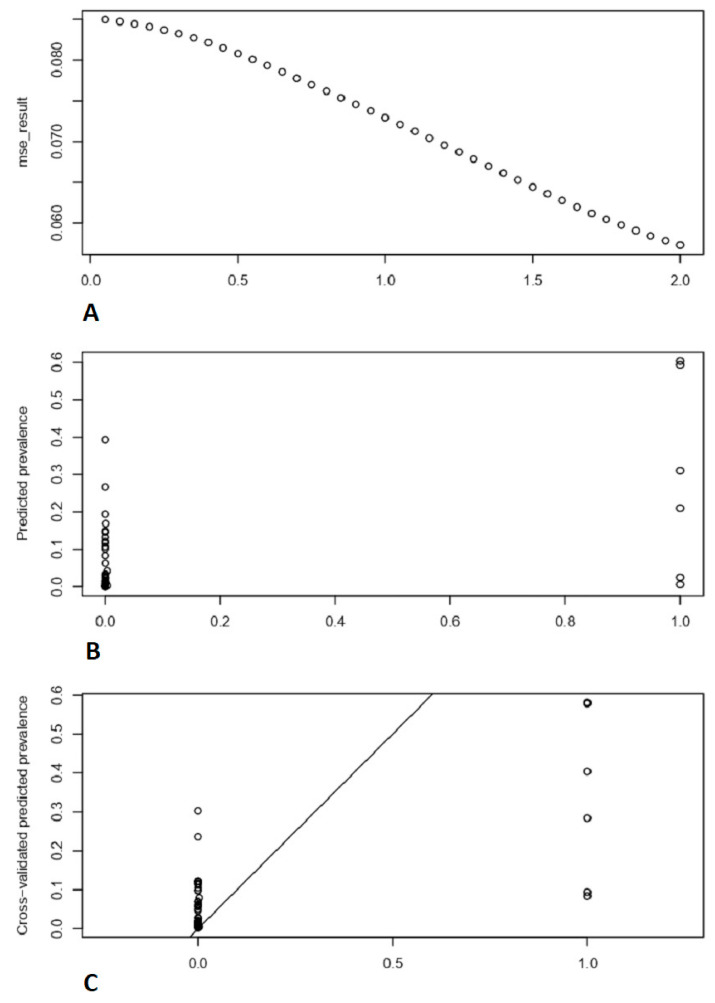
Depicts the comparison of observed prevalence rate with cross validated predicted prevalence rate. (**A**) Mean squared error against different powers. (**B**) “Predicted prevalence” vs. “observed prevalence”. (**C**) “Cross-validated predicted prevalence” vs. “observed prevalence”.

The powers with mean standard errors graph and with optimal power observed prevalence vs. predicted prevalence are shown in Figure 4.

The minimum ‘MSE’ was recorded at power = ‘2′. To simulate spatial interaction between locations, an exponential function altering the distance weight is frequently utilized. When using the IDW approach to anticipate unknown attribute values at specific places, such a function is frequently used [30].

In addition to IDW predictions, we used the kriging approach to predict the point prevalence rate of HPAI epidemics. Initially, before kriging, we plotted the known point prevalence rate data against x and y coordinates and examined the data to see if a first order trend was evident [54]. There was no such trend observed in the data, as can be seen in a summary plot of the point prevalence against the x and y coordinates depicted in Supplementary Figure S1 (see Supplementary Materials).

Whenever there is a spatially correlated distance or directional bias in the data, kriging is the best method to use [55]. For examining spatial variability in the data, a variogram model was used.

**Figure 5:**
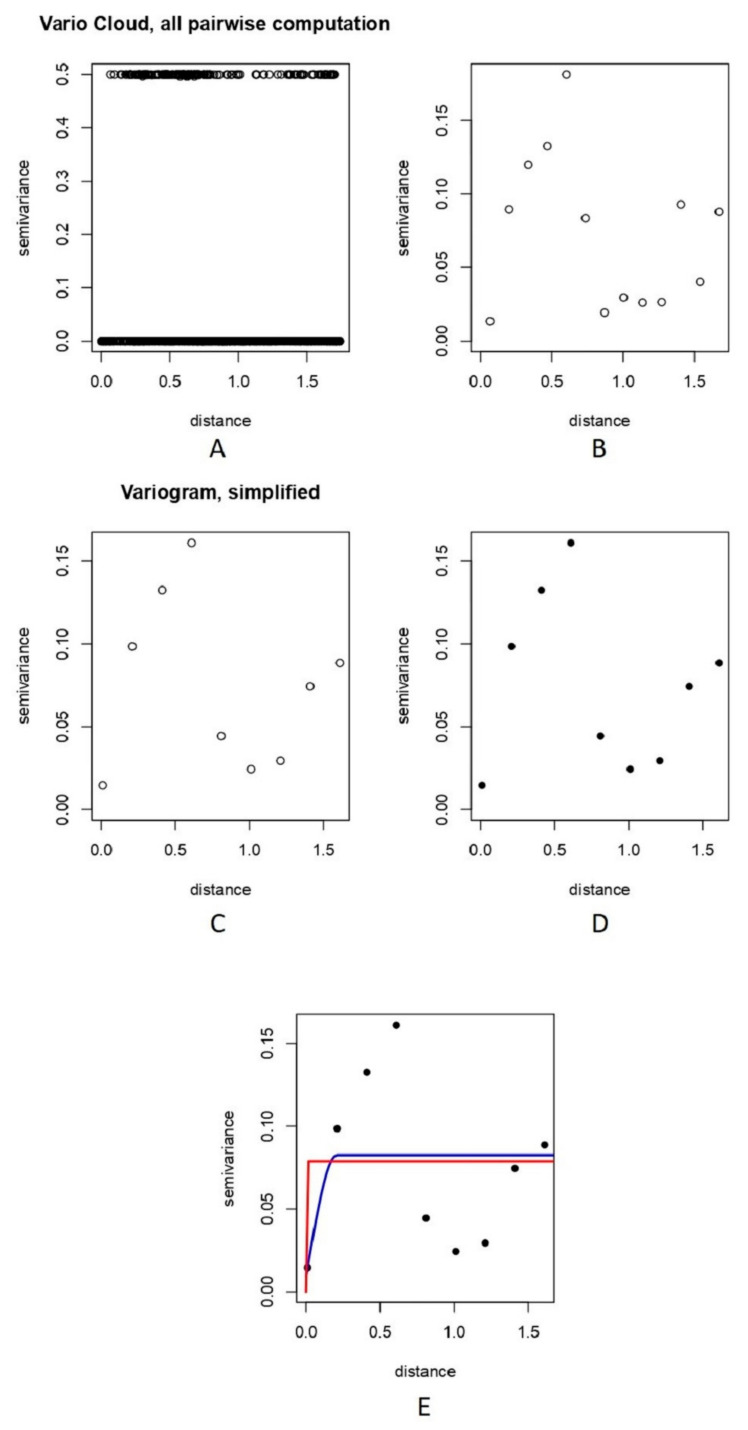
Showing a measure of variability between pairs of points at various distances. (**A**) A variogram displayed as a “vario cloud”. (**B**) Bin points by distance using 2nd order trend, depicting relationship between semi-variance and distance class. (**C**) Simplified variogram depicting relationship between semi-variance and bin points at distances using decimal degrees. (**D**) Variogram binning at 0.2 decimal degrees. (**E**) The blue line indicates spherical model fit, while red line indicates exponential model fit.

A variogram was obtained by restricting variogram estimates to half the maximum interpoint distance, which can be seen in Figure 5A.

The vario cloud from Figure 5A essentially illustrates clustering. Values that are more similar (i.e., those that have lower semi-variance) are also closer together (i.e., are in smaller distance classes). Values that are more different (i.e., have higher semi-variance) are also farther apart (i.e., are in higher distance classes).

The vario cloud version of the variogram, as shown in Figure 5A, is a bit intimidating to look at; it displays all values on the distance matrix as well as all semi-variance values.

A variogram generated after adding “Trend = 2nd” to the plot command can be seen in Figure 5B,C. Another variogram plotted by trying bins of 0.2 decimal degrees is shown in Figure 5D. The selection of a variogram model can alter the outcomes of an interpolating predictive assessment and can be used to demonstrate how a best-fitted model can be chosen [56]. Arétouyap et al. [56] further illustrated that the variogram model chosen has an unavoidable impact on the outcomes of kriging at both ends and the magnitude of the predicted value range. Spherical and exponential models to fit the variogram are shown in Figure 5E.

Examining the summaries of the spherical and exponential models and also the lower sum of squares indicated the spherical model to be the best fit to our data (see Supplementary Tables S1 and S2). Variogram analysis is quite well-acknowledged for being a valuable method for describing spatial data and for measuring spatial autocorrelation between sample points [57].

**Figure 6:**
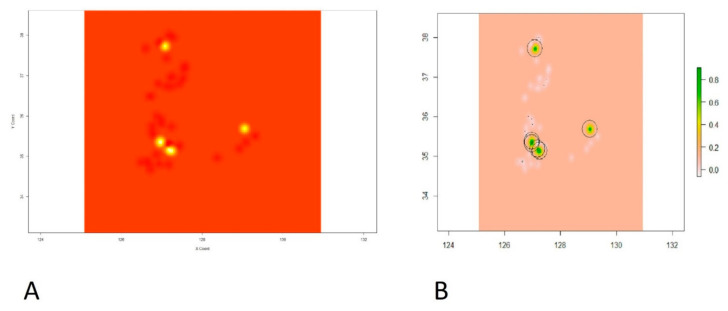
Showing the intensity of the point prevalence rate on xy coordinate plane. (**A**) Kriged predictions of HPAI H5N8 epidemic second phase in the coordinate plane indicating longitude on X-axis and latitude on Y-axis. (**B**) Prediction raster with colored scale from gray to yellow and green on the right panel indicating the intensity of the point prevalence rate. The green color shows the highest predicted point prevalence rate followed by yellow, gray, and then white for the lowest point prevalence rate.

Based on the best fit spherical model, the kriging method was applied, and point prevalence rate predictions were visualized, which can be seen in Figure 6. Kriged predictions and raster images of our predictions are shown in Figure 6A and Figure 6B, respectively.

**Figure 7:**
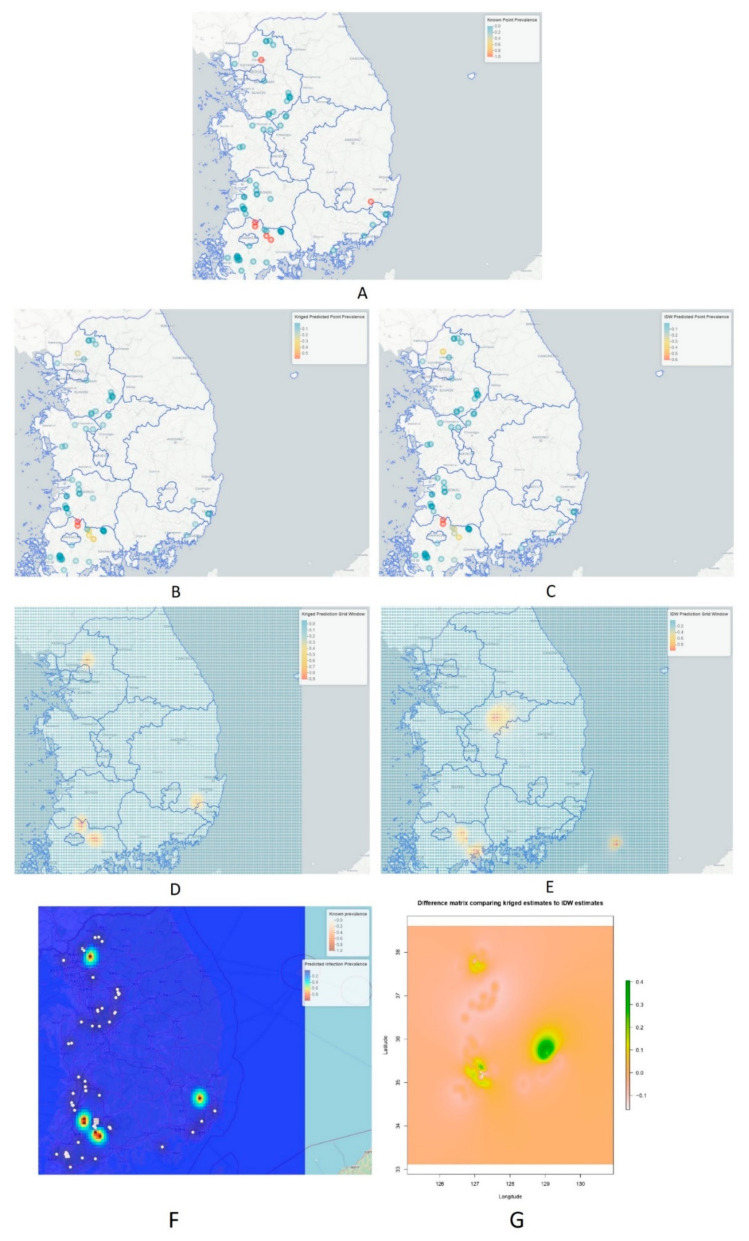
Showing the point prevalence rate of HPAI H5N8 during 2nd phase, also depicting risk predictions based on kriging and IDW methods. (**A**) Known point prevalence rate during second phase of HPAI H5N8 epidemic. (**B**) Kriged prediction from the points where data were collected. (**C**) IDW predictions from the points of collected data. (**D**) Kriged prediction for the whole prediction grid window. (**E**) IDW prediction for the whole prediction grid window. (**F**) Comparison of known prevalence and kriged predicted prevalence with scale bar on the upper right corner. (**G**) The difference between using IDW versus kriging to interpolate our point prevalence data. Regions of greatest difference are indicated in green.

**Figure 8:**
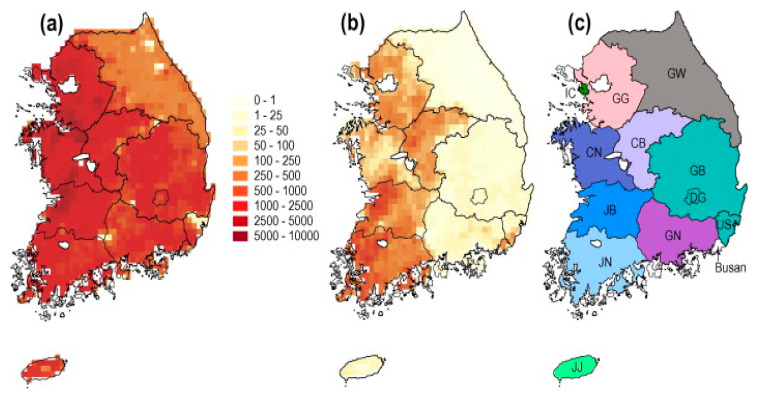
Maps showing domestic poultry density (number per square kilometer, colors in key) in ROK according to Gridded Livestock of the World 2.0 (Robinson et al., 2014). (**a**) Domestic chicken density. (**b**) Domestic duck density. (**c**) Map of provinces. Colors correspond to the branch color scheme used in Figure 3. Province abbreviations are as follows; CB: Chungbuk, CN: Chungnam, DG: Daegu, GB: Gyeongbuk, GG: Gyeonggi, GN: Gyeongnam, GW: Gangwon, IC: Incheon, JB: Jeonbuk, JJ: Jeju, JN: Jeonnam, US: Ulsan. (For interpretation of the references to colors in this figure legend, the reader is referred to the web version of this article.).

Finally, the predictions were depicted on the map for “IDW” and “Kriging” using leaflet, as visualized in Figure 7. The predictions were compared with maps downloaded from an online source, depicting duck population density and chicken population density in various parts of the country (South Korea), as shown in Figure 8 [16,58].

The mean squared error, called ‘MSE’, for the IDW technique was 0.05730333, whereas the ‘MSE’ for kriging was 0.04980171. For our study, the performance of kriging was shown to be slightly more preferable [59], but it may be dependent on the structure of the data [34,60]. A study on comparative analysis of interpolation methods indicated that the amount of bias in estimating is lowest for kriging and largest for IDW, as indicated by the corresponding MEs, based on the prediction errors [34]. It can be observed that IDW predicted some areas of high-risk point prevalence rate outside the range of the sampled datapoints (see Figure 7E).

## 4. Discussion

From the results of Figure 7D,F,G, it can be observed that kriged predictions showed high predicted risk for HPAI H5N8 epidemic in the nearby surrounding areas of Damyang-gun, Jeollanam-do province, with some areas of Sunchang-gun, Jeollabuk-do, followed by Uijeongbu-si, Gyeonggi-do province as well as the areas along the borderline where Gyeongsakbuk-do, Ulsan and Gyeongsangnam-do provinces converge. According to Hill, S.C. et al. [16,58], with the exception of Gangwon province, other provinces, e.g., Gyeonggi-do, Chungcheongnam-do, Chungcheongbuk-do, Jeollabuk-do, Ulsan and Busan have greater duck and chicken population densities, which can be seen in Figure 8 downloaded from an online source. Duck poultry do not typically exhibit clinical signs of HPAI infections; however, these birds aid in the propagation of HPAI infections into the chicken population [25]. As a result, it is possible to assume that duck populations cause the emergence and spread of H5N6 in the locations identified as hotspots, where these birds caused large infections and death in chickens during the H5N6 pandemic [16]. Comparatively, IDW predictions were similar to the kriged prediction when predictions were applied to the points from where data was collected, which can be observed in the known point prevalence map in Figure 7B,C, but were found to be different in some regions when predictions were applied for all the locations including the areas from where no data were collected, as shown in Figure 7E (xy coordinate prediction grid). IDW predicted higher risk in Seokgok-myeon, Gokseong-gun, Jeollanam-do, Suncheon-si, Jeollanam-do, Chungju-si, and Chungcheongbuk-do. In comparison to all other provinces in the Republic of Korea, Chungcheongbuk-do, Jeollabuk-do province and Jeollanam-do provinces have a greater domestic duck poultry density as well as chicken density (number per sq. km) [16,61]. From Figure 7G, it can be observed that kriged predictions were mainly different in Jeollanam-do province, Gyeonggi-do province, and mainly in the areas where Gyeongsakbuk-do, Ulsan and Gyeongsangnam-do provinces meet, which are depicted as green color spots on the difference matrix comparing kriged estimates to IDW estimates. According to Kwon et al. [15], a higher density of domestic ducks might lead to a higher number of avian influenza outbreaks. Moreover, districts in Jeollabuk-do province, being nearer to the Yellow Sea, might be comparatively more at risk, as water bodies can be hazardous for avian influenza spread [62,63,64].

Our study had some limitations: In Korea, HPAI reporting, and depopulation are completed rapidly, preventing adjacent disease spread (through anticipatory depopulation) from affected farms to surrounding chicken farms. In addition, if the government’s response to farm reporting is not optimal, there may be circumstances when notifications of the existence of HPAI H5N8 are not issued. In the case of HPAI H5N8 in Korea, infected ducks did not display clinical indications, which might have complicated H5N8 diagnosis [28,35].

In this study, variables contributing to the risk of HPAI outbreaks, e.g., poultry density, duck density, agricultural population density, forestry density, and proportion of land covered by surface water, were not assessed. Further research can evaluate these variables in the context of spatial and temporal interactions to provide a more thorough study of the risk factors generating HPAI epidemics. Furthermore, collecting samples from diverse species in the provinces for a more complete examination regarding rodents’ as well as wild birds’ involvement in the transmission of HPAI viruses is highly advised.

## 5. Conclusions

The high-risk districts including Damyang-gun, Jeollanam-do province with some areas of Sunchang-gun, Jeollabuk-do, Uijeongbu-si, Gyeonggi-do province, as well as the areas along the borderline where Gyeongsakbuk-do, Ulsan and Gyeongsangnam-do provinces converge, should apply specific biosecurity measures prior to birds’ migration periods. Strong biosecurity measures enhancing the cleanliness of the poultry surroundings will definitely help in the prevention of the spread of HPAIV into poultry farms. Therefore, poultry farms should strictly adhere to all applicable biosecurity measures to avoid any direct or indirect contact with surrounding inland water bodies. Such measures include using nets on farms, barricading all entry points to farm premises, properly fumigating and disinfecting areas inside and outside farms (particularly areas surrounding water bodies), covering stored water, properly disinfecting nearby stagnant waters, and restricting the entry of visitors and vehicles. Most importantly, it is strongly advised that proper personal sanitary practices be regularly followed when entering poultry farms. During the migration period of wild birds, spraying the areas surrounding inland water bodies with proper disinfectants is strongly recommended.

## Data Availability

The datasets that corroborate the conclusions of this study are available from the corresponding author upon reasonable request.

## Supplementary Materials

The following are available online at https://www.mdpi.com/article/10.3390/vetsci9030139/s1, Table S1: Summary of spherical variogram model to best fit the data, Table S2: Summary of Exponential variogram model, Figure S1: Shows the the trend in the point prevalence data; Algorithm S1: R.Script.
