# Supplementary material for: Interpolation of Point Prevalence Rate of the Highly Pathogenic Avian Influenza Subtype H5N8 Second Phase Epidemic in South Korea
Source: Vet Sci. 2022 Mar 16;9(3):139. doi: 10.3390/vetsci9030139 (PMC8954420; doi:10.3390/vetsci9030139)
Supplement: Supplementary file 1 [file vetsci-09-00139-s001.zip › Supplementary Figure and Tables.pdf]

Supplementary Figure 1. Shows the the trend in the point prevalence data

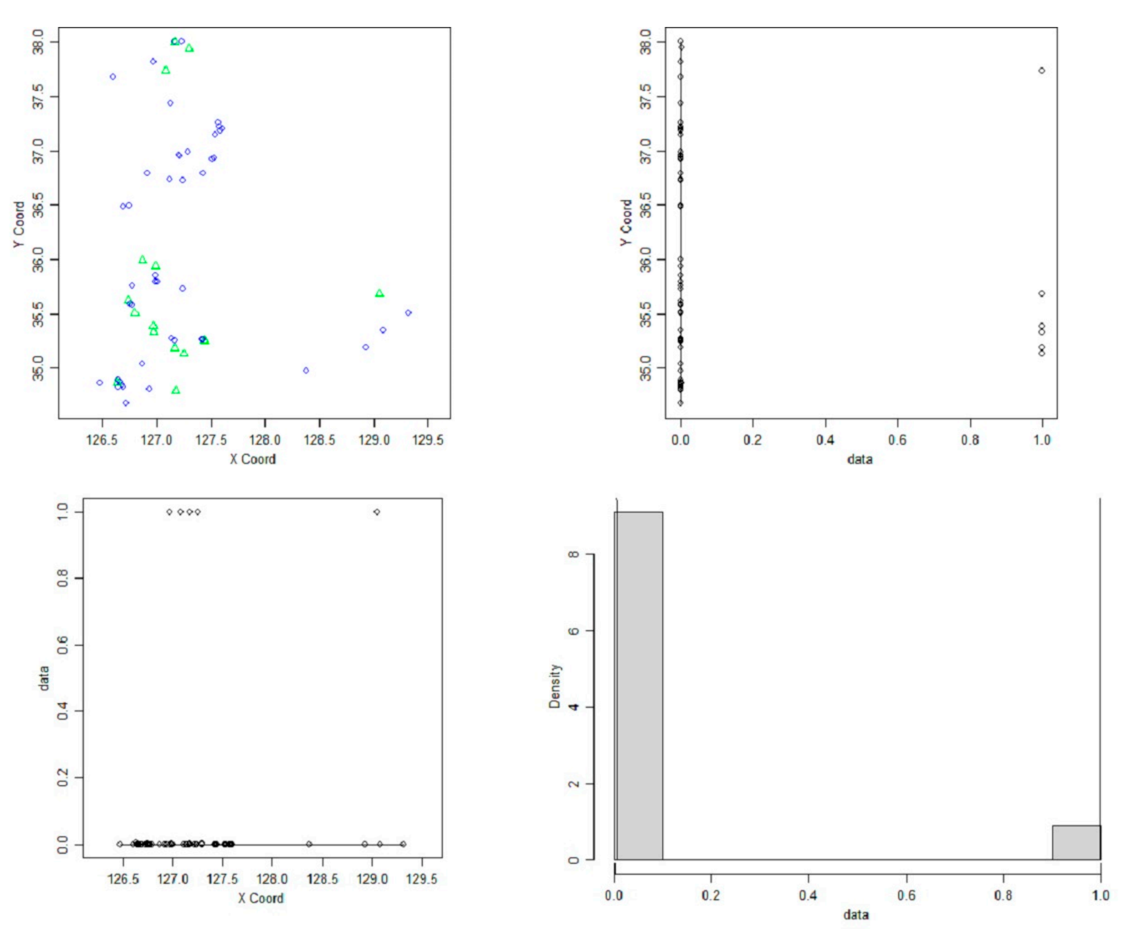

Supplementary Table S1. Summary of spherical variogram model to best fit the data

summary(VarioMod_sph)
$pmethod<br>[1] "WLS (weighted least squares)"
$cov.model<br>[1] "spherical"
$spatial.component<br>sigmasq phi<br>0.07330318 0.20875158
$spatial.component.extra<br>kappa<br>0.5
$nugget.component<br>tausq<br>0.009230274

$fix.nugget<br>[1] FALSE
$fix.kappa<br>[1] TRUE
$practicalRange<br>[1] 0.2087516
$sum.of.squares<br>value<br>2.745433
$estimated.pars<br>tausq sigmasq phi<br>0.009230274 0.073303184 0.208751579
$weights<br>[1] "npairs"
$call<br>variofit(vario = Vario, cov.model = "sph")
attr(,"class")<br>[1] "summary.variomodel"

Supplementary Table S2. Summary of Exponential variogram model

summary(VarioMod_exp)<br>$pmethod<br>[1] "WLS (weighted least squares)"
$cov.model<br>[1] "exponential"
$spatial.component<br>sigmasq phi<br>0.0788204422 0.0007487053
$spatial.component.extra<br>kappa<br>0.5
$nugget.component<br>tausq<br>0
$fix.nugget<br>[1] FALSE
$fix.kappa<br>[1] TRUE
$practicalRange<br>[1] 0.00223611
$sum.of.squares<br>value<br>3.047481
$estimated.pars

tausq sigmasq phi<br>0.0000000000 0.0788204422 0.0007487053
$weights<br>[1] "npairs"
$call<br>variofit(vario = Vario, cov.model = "exp")
attr(,"class")<br>[1] "summary.variomodel"
